# Research protocol for a systematic review and meta-analysis of the effects of music on anxiety and physiological outcomes in patients undergoing bronchoscopy

**DOI:** 10.1371/journal.pone.0313833

**Published:** 2025-01-07

**Authors:** Amani Kacem, Dhekra Chebil, Sana Aissa, Anis Maatallah, Ahmed Abdelghani

**Affiliations:** 1 Pulmonology Department, Ibn El Jazzar University Hospital, Faculty of Medicine of Sousse, University of Sousse, Kairouan, Tunisia; 2 Preventive Medicine Department, Ibn Al Jazzar University Hospital, Faculty of Medicine of Sousse, University of Sousse, Kairouan, Tunisia; 3 Pneumology Department, University Hospital Farhat Hached, Research Laboratory LR—Interaction Cœur-Poumons (LR14ES05), Faculty of Medicine of Sousse, University of Sousse, Sousse, Tunisia; European Institute of Oncology: Istituto Europeo di Oncologia, ITALY

## Abstract

**Introduction:**

Bronchoscopy is a routine clinical examination that can cause discomfort and anxiety in patients. This paper presents a protocol for a systematic review and meta-analysis aiming to assess the effect of music on anxiety and physiological outcomes in patients undergoing bronchoscopy.

**Methods:**

The protocol adhere to Preferred Reporting Items for Systematic Reviews and Meta-Analyses Protocols guidelines and has been registered in PROSPERO (CRD42024567398). Our documentary research strategy will involve four databases: PubMed, Google Scholar, Scopus, and the Cochrane Library. In addition, manual searches will be conducted through related articles and references. We will include randomized controlled trials that evaluate the effect of music on patients undergoing bronchoscopy. The primary outcome will be the anxiety level and the secondary outcome will include physiological outcomes. Study selection, data extraction, and quality assessment will be carried out independently by two reviewers. Any discrepancies will be resolved through consultation with a third reviewer. The quality and the risk of bias in the studies will be evaluated using The Joanna Briggs Institute critical appraisal tool. The results of this systematic review will be synthesized to provide an overview on the effectiveness of music on anxiety and physiological parameters in patients during bronchoscopy. If the results are considered acceptable and sufficiently homogeneous, a meta-analysis will be performed to synthesize the findings.

**Conclusion:**

The systematic review produced from this protocol will provide evidence on the effectiveness of music for patients undergoing bronchoscopy and will contribute to strengthening the existing body of knowledge on non-pharmacological interventions for anxiety management during medical procedures.

## Introduction

Bronchoscopy is a widely used procedure in pneumonology with interest for both diagnostic and therapeutic purposes [1]. Despite its safety and lack of major complications, patients frequently experience stress, discomfort, and anxiety during the procedure, which can negatively impact its success [2]. These feelings often lead to physiological changes, such as shortness of breath, tachycardia, and increased blood pressure [3, 4], further compromising patient comfort and increasing the complication rate [5]. Anxiety during endoscopic procedures typically stems from fear of the unknown, anticipation of pain and discomfort, concerns about the diagnosis or prognosis of underlying conditions, and a perceived lack of control [6, 7].

While pharmacological treatments like sedative drugs can reduce anxiety [8], they come with high costs and significant side effects, including cardiorespiratory issues like hypotension, tachycardia, and respiratory depression, as well as digestive problems like nausea and vomiting [9]. In recent years, complementary medicine has introduced musical interventions as an effective and inexpensive option for reducing anxiety during medical procedures. Listening to music before and during stressful medical interventions has shown significant positive changes in cortisol levels [10], implicating the endocrine and autonomic nervous systems in the response [11]. Music can trigger cognitive processes linked to stress in the brain, influencing psychological responses and reducing perceived stress and anxiety [12]. By providing a sense of control and enhanced coping ability, music helps patients alter their perception of negative sensations [13, 14]. Numerous experimental designs have evaluated the benefits of music during medical and surgical interventions across various healthcare settings [15]. Systematic reviews have synthesized these findings, particularly in the context of intensive care [16], coronary care [17], perioperative areas [18], cancer care [19], and maternity units [20].

Despite the growing body of evidence supporting music intervention, few RCTs have specifically assessed its effect during bronchoscopy. Interest in this area has grown, leading to further studies with conflicting results [21]. These studies often have small sample sizes and inconclusive results [22], highlighting the need for more comprehensive research. Although a systematic review on the effects of music during bronchoscopy was published in 2016, New randomized controlled trials (RCTs) [23–25] have been conducted since then, providing additional data and updated perspectives. The earlier review laid an important foundation of knowledge, but it did not account for more recent studies that could alter or refine the existing conclusions. Furthermore, the 2016 systematic review did not specifically focus on the impact of music on anxiety, which is a critical aspect of patient well-being during invasive procedures such as bronchoscopy. Therefore, a new systematic review is warranted to incorporate these recent studies and to focus more closely on anxiety as the primary outcome, which could have significant clinical implications for enhancing patient comfort and satisfaction.

The aim of this research protocol is to outlines the methodological approach for conducting a systematic review and meta-analysis aiming to assess the published evidence about the effects of music on anxiety and physiological parameters during bronchoscopy.

## Methods

This review protocol has been registered in the International Prospective Register of Systematic Reviews (PROSPERO) under the reference code CRD42024567398.The protocol is reported in accordance with the Preferred Reporting Items for Systematic Reviews and Meta-analysis Protocol (PRISMA-P) guidelines [26] **(S1 Checklist).** The final report will adhere to the Preferred Reporting Items for Systematic Reviews and Meta-Analyses (PRISMA) guidelines: the PRISMA statement [27].

### Review question

The systematic review and meta-analysis will seek to address the following main research question: Does music have impact on the anxiety and physiological outcomes of patients undergoing bronchoscopy?

### Eligibility criteria

The research question is formulated using the PICOS (Population, Intervention, Comparison, Outcome, Study design) model [28]. The eligibility criteria for study inclusion will be defined based on the PICOS framework as follows:

- Population (P): Adult patients aged 18 years or older undergoing bronchoscopy.
- Intervention (I): studies involving the use of music before and/or during bronchoscopy as an intervention regardless of the type of music or method of delivery will be included.
- Comparison (C): Comparative groups will include those receiving standard care without a music intervention or another non-pharmacological intervention.
- Outcome(O): The primary outcome of interest will be the level of patient anxiety as measured by the State-Trait Anxiety Inventory (STAI) or other validated scales (such as Visual Analog Scale (VAS) and physiological outcomes such as heart rate (HR), arterial pressure, oxygen saturation (O₂ sat), and pain score.
- Study design (S): RCTs published in any language will be included. Only completed RCTs will be included. There will be no restrictions on the study setting, country of origin, or publication date.

Studies will be excluded if the intervention involves another non-pharmacological method in addition to music or if they assess multimodal interventions where the specific effect of music cannot be isolated. Additionally, quasi-experimental studies, observational studies, case studies, editorials, letters to the editor, and conference abstracts will be excluded. Studies without a comparison group or without at least one measurement of pain score, anxiety score, or physiological parameters will also be excluded.

### Search strategy

An electronic search will be conducted across four reputable databases: Medline via PubMed, google scholar, Scopus and the Cochrane library, from their inception. The search will be updated periodically to ensure all relevant articles are included before finalizing the study results. We developed search strategies using a combination of Medical Subject Headings (MeSH) terms and synonyms related to music, bronchoscopy, anxiety and physiological parameters and tailored to each specific database **(Table 1).** Boolean operators ‘AND’ and ‘OR’ will be used.

**Table 1:** Search terms.

Search Term	Query
Music	"Music" OR "Music Therapy"
Bronchoscopy	"Bronchoscopy"
Anxiety	"Anxiety" OR "Stress, Psychological" OR "Anxiety Disorders" OR "Pain" OR " patient comfort"
Physiological Parameters	"Heart Rate" OR " pulse rate" OR " cardiac rate" OR "Blood Pressure" OR " arterial pressure" OR " diastolic pressure" OR " systoloic pressure" OR "Oxygen Saturation" OR " blood oxygen level"
Randomized Controlled Trial	"Randomized Controlled Trial"

The preliminary PubMed search strategy is as follows:

(music OR music therapy) AND (bronchoscopy) AND ((anxiety OR stress, psychological OR anxiety disorders) OR (pain OR patient comfort) OR (heart rate OR pulse rate OR cardiac rate) OR (blood pressure OR arterial pressure OR diastolic pressure OR systolic pressure) OR (oxygen saturation OR blood oxygen level) OR (vital signs)). this search query will be adapted to the other databases. The complete database search strategy is presented in **S1 Appendix**. In Google scholar, the search results will be sorted by relevance and only the first 10 pages of the search results will be examined using the same terms.

In addition to searching electronic databases, the reference lists of all included articles will be carefully examined to uncover any additional relevant studies.

### Study selection

**Fig 1:**
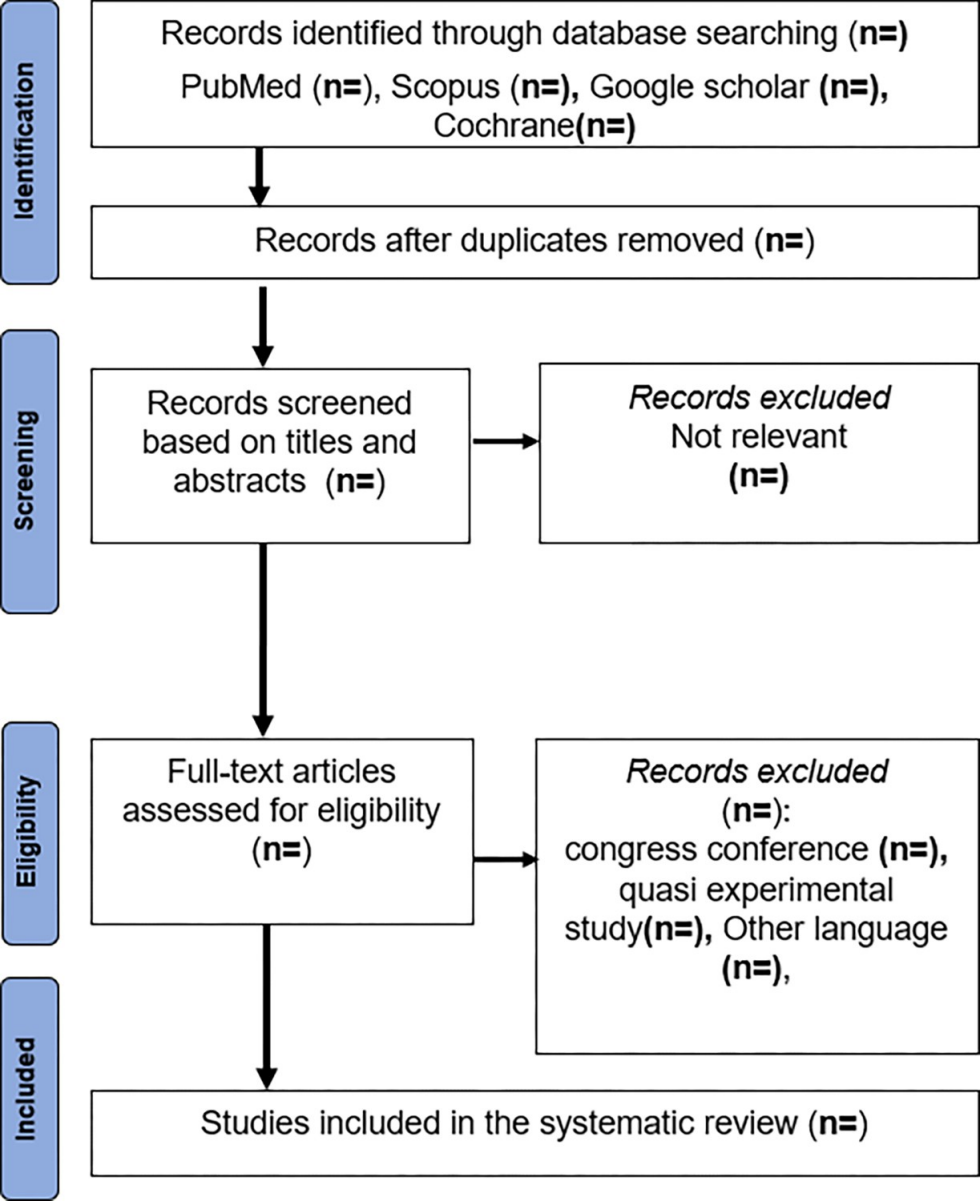
Flow diagram of study selection.

The search results from each database will be combined and duplicates will be removed using Zotero bibliographic software. Studies will then be screened independently by two reviewers (***AK*** and ***DC*** in the authors’ list). Any disagreements will be discussed, and if a consensus could not be reached, a third reviewer (SA in the authors’ list) will be consulted. Initially, the titles of the studies will be independently screened by the two reviewers to assess their relevance to the research question. Subsequently, the abstracts will be reviewed to ensure they meet the inclusion criteria. Articles with abstracts that satisfy these criteria will then undergo a thorough full-text review to determine their eligibility for inclusion in the systematic review. **The PRISMA flow diagram** will be used to provide a detailed description of the study selection process for the systematic review **(Fig 1).**

### Data extraction

Data will be meticulously extracted from all eligible studies by two independent investigators (***AK*** and ***DC*** in the authors’ list) using a predesigned data extraction form in Microsoft Excel to ensure precision and consistency. The extracted data will be then subjected to a thorough verification process by a third author (SA in the authors’ list) to ensure its accuracy and completeness. The following key information will be extracted from each study:

- Study Identification: First author, year of publication, country.
- Participants Characteristics: Demographic data such as age distribution, sex, and any inclusion/exclusion criteria applied.
- Study Design: Sample size, setting, and duration of follow-up.
- Intervention Details: Specifics of the music intervention, including type of music, duration, timing relative to the bronchoscopy procedure, delivery method, and any concurrent interventions.
- Comparison Group: Description of the control or comparison group (e.g., standard care, another non-pharmacological intervention).
- Outcomes: Primary and secondary outcome measures.
- Results: Key findings related to the impact of the music intervention on the specified outcomes.

After data extraction, one investigator will compile a summary of the data to consolidate the findings from the two investigators.

### Methodological quality assessment

To evaluate the methodological quality of the included studies, the Joanna Briggs Institute (JBI) critical appraisal tool will be used. This tool rates different parts of the study quality as "yes", "no", "unclear", or "not applicable." [29]. To ensure reliability, two investigators (AK and DC) will independently apply the JBI checklist to each study. Any discrepancies will be resolved through discussion, with a third investigator (SA). The percentage of affirmative responses to the checklist items (**Table 2**) will determine the final quality score for each study. Studies will then be classified into one of three categories based on their scores: "high risk of bias" (with affirmative responses up to 49%), "moderate risk of bias" (with affirmative responses ranging from 50% to 69%), or "low risk of bias" (with affirmative responses exceeding 70%) [30].

**Table 2:** JBI items.

Items	Yes	No	Not applicable	Unclear	Risk of bias
**Item 1**. Was true randomization used for assignment of participants to treatment groups?					
**Item 2**. Was allocation to treatment groups concealed?					
**Item 3**. Were treatment groups similar at the baseline?					
**Item 4**. Were participants blind to treatment assignment?					
**Item 5**. Were those delivering treatment blind to treatment assignment?					
**Item 6**. Were outcomes assessors blind to treatment assignment?					
**Item 7**. Were treatment groups treated identically other than the intervention of interest?					
**Item 8**. Was follow up complete and if not, were differences between groups in terms of their follow up adequately described and analyzed?					
**Item 9**. Were participants analyzed in the groups to which they were randomized?					
**Item 10**. Were outcomes measured in the same way for treatment groups?					
**Item 11**. Were outcomes measured in a reliable way?					
**Item 12.** Was appropriate statistical analysis used?					
**Item 13.** Was the trial design appropriate, and any deviations from the standard RCT design (individual randomization, parallel groups) accounted for in the conduct and analysis of the trial?					

If an article lacks or has insufficient data, basic information will be emailed to the relevant authors. Studies will be excluded if contact is not established, or the data is insufficient.

### Grading of evidence

The Grading of Recommendations Assessment, Development, and Evaluation (GRADE) method will be used to assess the quality of evidence for each intervention, rating it as high, moderate, low, or very low [31]. This process will be conducted via the GradePro software. A summary of findings table will present primary and secondary outcomes, along with the risk of publication bias. Evidence rated as low or very low will be highlighted in the conclusion, guiding careful clinical decision-making and interpretation of music’s effect on anxiety and physiological outcomes during bronchoscopy.

### Missing data

If an article lacks or has insufficient data, basic information will be emailed to the relevant authors. Data will be excluded from the quantitative analysis if contact is not established after two attempts, or if the data remains insufficient. However, if appropriate, an imputation strategy will be performed to handle missing data.

### Statistical analysis

Data from the included studies will be synthesized quantitatively using a random-effects model to account for variability both within and between studies. Effect sizes will be calculated for each outcome, such as anxiety levels and physiological parameters, and reported as standardized mean differences (SMD) with 95% confidence intervals (CIs). Heterogeneity across studies will be assessed using Cochran’s Q test and the I^2^ statistic, with I^2^ values of 25%, 50%, and 75% representing low, moderate, and high heterogeneity, respectively. If substantial heterogeneity is detected, and sufficient data are available, subgroup analyses based on age, sex ratio, in patient/outpatient, type of sedation and operator, will be conducted to explore potential sources of variability. Additionally, a sensitivity analysis will be performed to assess the robustness of the results. In cases where more than ten studies are included, publication bias will be evaluated using a funnel plot and Egger’s test. All statistical analyses will be performed using R software Version 4.4.1.

#### Sensitivity analysis

To assess the robustness of the meta-analysis findings, a sensitivity analysis will be conducted as part of the systematic review using one by one elimination. This analysis aims to evaluate how variations in study inclusion criteria, outcome measures, and methodological quality impact the overall results. Specifically, we will explore the effects of excluding studies with a high risk of bias, as well as those employing different types of music interventions or varying follow-up time of exposure to identify source of heterogeneity. Additionally, we will perform subgroup analyses based on demographic factors such as age and sex to determine if these variables influence the efficacy of music on anxiety and physiological parameters during bronchoscopy.

### Ethics and dissemination

The systematic review and meta-analysis will utilize publicly available literature and data. Hence, This research is exempt from ethical review. The results will be published in a peer-reviewed journal. Any changes to the protocol will be registered in the PROSPERO database (registration number CRD42024567398), and the updated version will accompany the final systematic review.

### Timeline

We expect to finalize the search, screening, data extraction, and synthesis processes by late September 2024.

## Discussion

This systematic review and meta-analysis will likely demonstrate whether music has a significant impact on reducing anxiety and improving physiological parameters in patients undergoing bronchoscopy. It is expected that music therapy, being a non-pharmacological intervention, could offer a valuable alternative to conventional sedation and anxiety management methods, reducing drug-related side effects.

What sets this review apart from previous studies is its exclusive focus on bronchoscopy and the inclusion of a detailed analysis of both psychological and physiological outcomes. This systematic review will also offer a comprehensive analysis using modern statistical methods with rigorous quality assessment through tools like GRADE. sensitivity analyses will also provide insights into the stability of our conclusions and help identify any potential confounding factors that may affect the interpretation of the impact of music therapy in this clinical context.

However, some limitations may arise, such as the limited number of completed clinical trials, small sample sizes, and heterogeneity among study designs and populations. These factors could limit the strength of the conclusions and the applicability of the findings to broader clinical settings. Additionally, the GRADE assessment may reveal that the certainty of the evidence is not high enough to immediately influence clinical guidelines.

## Conclusion

This systematic review will provide a comprehensive analysis of the available evidence on the effect of music as a non-pharmacological intervention on anxiety and physiological parameters during bronchoscopy, potentially offering an alternative to conventional anxiety management methods. The results could influence clinical guidelines and promote the integration of music into bronchoscopy practice.

## Supporting information

S1 Checklist PRISMA-P (Preferred Reporting Items for Systematic review and Meta-Analysis Protocols) 2015 checklist: Recommended items to address in a systematic review protocol. (DOCX)

S1 Appendix Detailed search strategy. (DOCX)

## References

[1] Qanash S, Hakami OA, Al-Husayni F, Gari AG. Flexible Fiberoptic Bronchoscopy: Indications, Diagnostic Yield and Complications. Cureus. 12(10):e11122. doi: 10.7759/cureus.11122 33133790 PMC7586410

[2] Kodalak Cengiz S, Yılmaz C, Yurdakul AS. The Role of Anxiety Scales and Serum Copeptin Levels in Determining the Preprocedure Anxiety Status of Patients Who Undergo Fiberoptic Bronchoscopy and Endobronchial Ultrasonography. Int J Clin Pract. 2024;2024(1):5524757.

[3] Lu G, Jia R, Liang D, Yu J, Wu Z, Chen C. Effects of music therapy on anxiety: A meta-analysis of randomized controlled trials. Psychiatry Res. 1 oct 2021;304:114137. doi: 10.1016/j.psychres.2021.114137 34365216

[4] Tamam MO, Bagcioglu E, Mulazimoglu M, Tamam L, Ozpacaci T. Evaluation of anxiety and depression in patients prior to myocardial perfusion scintigraphy. Int J Psychiatry Clin Pract. 1 juin 2012;16(2):93–7. doi: 10.3109/13651501.2011.631017 22136214

[5] Mitsumune T, Senoh E, Adachi M. Prediction of patient discomfort during fibreoptic bronchoscopy. Respirology. 2005;10(1):92–6. doi: 10.1111/j.1440-1843.2005.00642.x 15691244

[6] de L. Horne DJ, Vatmanidis P, Careri A. Preparing Patients for Invasive Medical and Surgical Procedures 1: Adding Behavioral and Cognitive Interventions. Behav Med. 1 mars 1994;20(1):5–13. 7919635 10.1080/08964289.1994.9934610

[7] Andrychiewicz A, Konarska K, Gorka K, Bartyzel S, Salek M, Biedron G, et al. Evaluation of factors that influence anxiety and satisfaction in patients undergoing bronchofiberoscopy with analgosedation. Clin Respir J. 2017;11(5):566–73. doi: 10.1111/crj.12384 26365048

[8] Prakash UBS, Offord KP, Stubbs SE. Bronchoscopy in North America: The ACCP Survey. Chest. 1 déc 1991;100(6):1668–75. doi: 10.1378/chest.100.6.1668 1959412

[9] Bell GD. Premedication, Preparation, and Surveillance. Endoscopy. 14 août 2002;34:2–12. doi: 10.1055/s-2002-19389 11778125

[10] Escher J, Höhmann U, Anthenien L, Dayer E, Bosshard C, Gaillard RC. [Music during gastroscopy]. Schweiz Med Wochenschr. 1 juill 1993;123(26):1354–8. 8393585

[11] Watkins GR. Music Therapy: Proposed Physiological Mechanisms and Clinical Implications. Clin Nurse Spec. mars 1997;11(2):43. doi: 10.1097/00002800-199703000-00003 9233140

[12] Thoma MV, Marca RL, Brönnimann R, Finkel L, Ehlert U, Nater UM. The Effect of Music on the Human Stress Response. PLOS ONE. 5 août 2013;8(8):e70156. doi: 10.1371/journal.pone.0070156 23940541 PMC3734071

[13] Burns J, Labbe E, Williams K, McCall J. Perceived and Physiological Indicators of Relaxation: As Different as Mozart and Alice in Chains. 10.1023/a:1023488614364 10652638

[14] Allen K, Golden LH, Izzo JLJ, Ching MI, Forrest A, Niles CR, et al. Normalization of Hypertensive Responses During Ambulatory Surgical Stress by Perioperative Music. Psychosom Med. juin 2001;63(3):487. doi: 10.1097/00006842-200105000-00019 11382277

[15] Rorke MA. Music Therapy in the Age of Enlightenment. J Music Ther. 1 mars 2001;38(1):66–73. doi: 10.1093/jmt/38.1.66 11407966

[16] Umbrello M, Sorrenti T, Mistraletti G, Formenti P, Chiumello D, Terzoni S. Music therapy reduces stress and anxiety in critically ill patients: A systematic review of randomized clinical trials. août 2019 [cité 1 août 2024]; Disponible sur: https://air.unimi.it/handle/2434/702084 10.23736/S0375-9393.19.13526-2 30947484

[17] Lieber AC, Bose J, Zhang X, Seltzberg H, Loewy J, Rossetti A, et al. Effects of music therapy on anxiety and physiologic parameters in angiography: a systematic review and meta-analysis. J NeuroInterventional Surg. 1 avr 2019;11(4):416–23. doi: 10.1136/neurintsurg-2018-014313 30415224

[18] Bradt J, Dileo C, Shim M. Music interventions for preoperative anxiety—Bradt, J—2013 | Cochrane Library. [cité 1 août 2024]; Disponible sur: https://www.cochranelibrary.com/cdsr/doi/10.1002/14651858.CD006908.pub2/abstract 10.1002/14651858.CD006908.pub2 PMC9758540 23740695

[19] Nightingale CL, Rodriguez C, Carnaby G. The Impact of Music Interventions on Anxiety for Adult Cancer Patients: A Meta-Analysis and Systematic Review. Integr Cancer Ther. 1 sept 2013;12(5):393–403. doi: 10.1177/1534735413485817 23625027

[20] Corbijn van Willenswaard K, Lynn F, McNeill J, McQueen K, Dennis CL, Lobel M, et al. Music interventions to reduce stress and anxiety in pregnancy: a systematic review and meta-analysis. BMC Psychiatry. 27 juill 2017;17(1):271. doi: 10.1186/s12888-017-1432-x 28750631 PMC5531014

[21] Harney C, Johnson J, Bailes F, Havelka J. Is music listening an effective intervention for reducing anxiety? A systematic review and meta-analysis of controlled studies. Music Sci. 1 juin 2023;27(2):278–98.

[22] Wang MC, Zhang LY, Zhang YL, Zhang YW, Xu XD, Zhang YC. Effect of music in endoscopy procedures: systematic review and meta-analysis of randomized controlled trials. Pain Med Malden Mass. oct 2014;15(10):1786–94. doi: 10.1111/pme.12514 25139786

[23] Jeppesen E, Pedersen CM, Larsen KR, Walsted ES, Rehl A, Ehrenreich J, et al. Listening to music prior to bronchoscopy reduces anxiety—a randomised controlled trial. Eur Clin Respir J. 2019;6(1):1583517. doi: 10.1080/20018525.2019.1583517 30915199 PMC6427702

[24] Jeppesen E, Pedersen CM, Larsen KR, Rehl A, Bartholdy K, Walsted ES, et al. Music does not alter anxiety in patients with suspected lung cancer undergoing bronchoscopy: a randomised controlled trial. Eur Clin Respir J. 2016;3:33472. doi: 10.3402/ecrj.v3.33472 27814780 PMC5097150

[25] Opartpunyasarn P, Vichitvejpaisal P, Oer-Areemitr N. The effect of binaural beat audio on anxiety in patients undergoing fiberoptic bronchoscopy: A prospective randomized controlled trial. Medicine (Baltimore). 17 juin 2022;101(24):e29392. doi: 10.1097/MD.0000000000029392 35713444 PMC9276398

[26] Shamseer L, Moher D, Clarke M, Ghersi D, Liberati A, Petticrew M, et al. Preferred reporting items for systematic review and meta-analysis protocols (PRISMA-P) 2015: elaboration and explanation. BMJ. 2 janv 2015;350:g7647. doi: 10.1136/bmj.g7647 25555855

[27] Liberati A, Altman DG, Tetzlaff J, Mulrow C, Gøtzsche PC, Ioannidis JPA, et al. The PRISMA Statement for Reporting Systematic Reviews and Meta-Analyses of Studies That Evaluate Health Care Interventions: Explanation and Elaboration. Ann Intern Med. 18 août 2009;151(4):W-65. 19622512 10.7326/0003-4819-151-4-200908180-00136

[28] Thomas J, Kneale D, McKenzie JE, Brennan SE, Bhaumik S. Determining the scope of the review and the questions it will address. In: Cochrane Handbook for Systematic Reviews of Interventions [Internet]. John Wiley & Sons, Ltd; 2019 [cité 1 août 2024]. p. 13–31. Disponible sur: https://onlinelibrary.wiley.com/doi/abs/10.1002/9781119536604.ch2

[29] Barker TH, Stone JC, Sears K, Klugar M, Tufanaru C, Leonardi-Bee J, et al. The revised JBI critical appraisal tool for the assessment of risk of bias for randomized controlled trials. JBI Evid Synth. 1 mars 2023;21(3):494–506. doi: 10.11124/JBIES-22-00430 36727247

[30] Ribeiro DM, Réus JC, Felippe WT, Pacheco-Pereira C, Dutra KL, Santos JN, et al. Technical quality of root canal treatment performed by undergraduate students using hand instrumentation: a meta-analysis. Int Endod J. mars 2018;51(3):269–83. doi: 10.1111/iej.12853 28862763

[31] Xie CX, Machado GC. Clinimetrics: Grading of Recommendations, Assessment, Development and Evaluation (GRADE). J Physiother. janv 2021;67(1):66. doi: 10.1016/j.jphys.2020.07.003 32859566

