# Supplementary material for: Research protocol for a systematic review and meta-analysis of the effects of music on anxiety and physiological outcomes in patients undergoing bronchoscopy
Source: PLoS One. 2025 Jan 7;20(1):e0313833. doi: 10.1371/journal.pone.0313833 (PMC11706486; doi:10.1371/journal.pone.0313833)
Supplement: S1 Appendix — (DOCX) [file pone.0313833.s002.docx]

**Detailed search strategy**

**For scopus:**

TITLE-ABS-KEY (music OR music therapy) AND (bronchoscopy) AND ((anxiety OR stress, psychological OR anxiety disorders) OR( pain OR patient comfort) OR (heart rate OR pulse rate OR cardiac rate) OR (blood pressure OR arterial pressure OR diastolic pressure OR systolic pressure) OR (oxygen saturation OR blood oxygen level) OR (vital signs))

**For cochrane**

(music OR music therapy) AND (bronchoscopy) AND ((anxiety OR stress, psychological OR anxiety disorders) OR( pain OR patient comfort) OR (heart rate OR pulse rate OR cardiac rate) OR (blood pressure OR arterial pressure OR diastolic pressure OR systolic pressure) OR (oxygen saturation OR blood oxygen level) OR (vital signs)):ti,ab,kw

**For pubmed**

(music OR music therapy) AND (bronchoscopy) AND ((anxiety OR stress, psychological OR anxiety disorders) OR( pain OR patient comfort) OR (heart rate OR pulse rate OR cardiac rate) OR (blood pressure OR arterial pressure OR diastolic pressure OR systolic pressure) OR (oxygen saturation OR blood oxygen level) OR (vital signs))
